# Notes and News

**Published:** 1887-11-12

**Authors:** 


### Notes and News.

Collected and Communicated.

Dr. W. C. Coles, of Bourton-on-the-Water, sends us the following, taken from "Gloucestershire Notes and Queries," vol. iii., p. 439 (London: W. Kent and Co., 23, Paternoster Row: "Extracts from Records of St. Peter's Hospital, Bristol.—A.D. 1698. An order given to purchase two iron chains, with locks, to fasten disorderly people to the blocks in purgatory—a place so called in the hospital—and also to erect a pair of stocks and a whipping-post in the yard. A.D. 1699. Ordered that the chirurgeons have £10 per annum paid them for their attendance in this house, and looking over the people who want their assistance, and likewise for medicines that shall be used in chirurgery, and also for shaving the men and cutting the boys' hair. A.D. 1702. The guardians agree with Mrs. Sarah Case for the cure of eleven scruffy heads of the boys, at five shillings each. A.D. 1703. Ordered, that the several persons under the care of this corporation now afflicted with the 'King's Evil' (not exceeding the number of twelve) be sent to Bath, at the charge of the corporation, in order to have a touch from the Queen (Anne) for a cure; and that the surgeons do nominate such as are most afflicted therewith, one of the surgeons to accompany them."

We regret to hear that the Crown Princess has telegraphed for Sir Morell Mackenzie, grave symptoms having again appeared in the throat of the Crown Prince, and it is feared he may have to undergo another operation.

Sister Nina Lee writes: "Not only one, but I may say, more than a hundred nurses and sisters, think it would be good if nurses' hours of work could be shortened. The very bravest and strongest workers in our hospitals grow weary at times. Why do our doctors and matrons not recognise this truth more than they do? It is a common cry, 'I am too tired to go out.' Who but a nurse knows the mental and physical strain of her work? of the weary, tired feet of the 'probationer'? Why should this be? A nurse's life is one of noble self-denial, a ministry of love for others; and yet how many nurses are compelled to take up other work because the strain is too much for them, the hours too long, and the pay so small? Pages might be written on the brave and noble deeds of some of the women who daily minister to the sick ones in our hospitals. Instances I have known when the sister has given up her night's rest to relieve her patient and help her nurse. E. A. S. remarks that one great thing so sadly lacking in hospital life is consideration for the workers, and it is so."

The Fifth of November brings with it a special set of accidents from the discharge of fireworks. One of these, which took place about a week before the "festival of Guy Fawkes," may, perhaps, serve as a warning to amateur pyrotechnists. Two boys bought a "maroon," a kind of firework which consists of a charge of gunpowder enclosed in a paper case, and bound round with string, in order to add all possible noise to the explosion. Having no match, they asked a passer-by to give them one. He had none, but readily offered to light the fuse of the maroon at the pipe he was smoking, the result being that it burst in his face, tearing up his cheek and seriously injuring one eye, while the two boys also were burnt and cut about the face. The maroon bore a printed label, stating that the fuse should not be ignited within five feet of any person, but this warning seems to have been disregarded. Surely some restriction should be placed on the sale of such dangerous toys to persons too young or too ignorant to use proper care in setting fire to them.

The following vacancies are announced this week, the amount of salary in each case being given after the name of the institution :—

**Resident Medical Officers.**

Birmingham General Dispensary.—£150.
Carnarvon Infirmary, Bangor. House Surgeon.—£100.
Central London Sick Asylum, Cleveland Street. Assistant Officer and Dispenser.—£100.
Lincoln County Hospital. House Surgeon.—£100.
Liverpool Infirmary for Children.—No salary.
Nottingham General Hospital. Surgical Assistant.—No salary.
Royal Albert Asylum, Lancaster. Assistant Medical Officer.—£120.
Royal Hospital for Diseases of the Chest, City Road. Junior House Physician.—£50.
Royal United Hospital, Bath. Resident Medical Officer.—£100.
Stroud General Hospital. House Surgeon.—£80.
Sussex County Hospital, Brighton. Assistant House Surgeon.
West Kent General Hospital, Maidstone. House Surgeon.—£150.
Wilts County Hospital, Devizes. Second Assistant.—£100.

**Non-resident Medical Officers.**

Bristol Royal Infirmary. Obstetric Physician.
Queen's College, Birmingham. Lecturer in Operative Surgery.
Queen's College, Birmingham. Professor of Gynæcology.
Sussex County Hospital, Brighton. Assistant Physician.
Western General Dispensary, Marylebone Road. Hon. Dental-Surgeon.
Westminster Hospital Medical School. Lecturer on Physiology.

**Matron.**

East Suffolk and Ipswich Hospital.—£60.
The Civil Hospital, Gibraltar. Apply to the Crown Agents, Downing Street.

**Nurses.**

Chelsea Hospital for Women. Charge Nurse.—£25, uniform and washing.
East Dulwich Grove New Infirmary. Ward Nurses.—£20 to £25, uniform and washing.
South Shields Union. Assistant, trained.—£25, washing, &c.
Liverpool Smithdown Road Workhouse. Three Nurses.—£25, washing, &c.
Glasgow Maternity Hospital. Want Nurses to train.
Croydon Nurses Institute.—£20, uniform.
Institution of Trained Nurses, Leicester. Medical, Surgical, and Monthly.

**Probationer.**

Oldham Infirmary. Probationer.

We have a science for everything nowadays. Even mirth has its method, and, apparently, its madness. At least that is what we gather from a lecture on "The Psychology of Joking," recently delivered by Dr. Hughlings Jackson to the London Medical Society. According to Dr. Jackson, a punster is nearly akin to a lunatic. We hope this severe judgment applies only to chronic sufferers from the malady of punning, and that the occasional aberrations to which we are all liable are to be regarded less severely. The object of a pun, he explains, is to give the "sensation of complete resemblance with a sense of vast difference," which is an absolute reversal of the true process of thought; which tends to tracing the relations of real likeness and unlikeness in different objects. The pun brings out only superficial and illusory resemblances, and the mental topsey-turveydom belonging to the habit of thought which produces it is akin to many of the delusions of the insane—for example, to that of the lunatic, who fancies that his body is a teapot, attitudinizes to suit his notion, and tries to pour tea from the spout arm. Looked at from this point of view, a joke is a very serious matter indeed; and doubtless after listening to Dr. Jackson's lecture his hearers determined to follow Lewis Carroll's advice, and shun—

"The shameless and abandoned one
Who stoops to perpetrate a pun."

Admiral White, C.B., presided at the annual meeting of the governors of the West of England Eye Infirmary at Exeter. General Drew was unanimously elected President for the ensuing year. The report stated that there had been a slight falling-off in the subscriptions, which had been more than counterbalanced by increased benefactions.

The sum of £14,000 is to be spent on enlarging the Fever Hospital at King's Cross, near Dundee.

The new cottage hospital at Faversham is all but completed, and a meeting was held on Tuesday, under the presidency of the Mayor (J. N. Goldfinch, Esq.), for the purpose of considering a series of rules for the management of the institution. The Vicar (the Rev. C. E. Don) and many of the principal inhabitants were present.

"Telephone" writes to the *Hastings Observer* to point out the various advantages of having the local hospital connected with the telephone exchange. This is a hint worthy of consideration by hospital authorities generally.

Although the Huntley Cottage Hospital has not got beyond the stage of the selection of a site, the gentlemen who are promoting it have met for the consideration of a constitution. Have they forgotten that rule of cookery which commences: "First catch your hare?"

Dr. Duncan Burgess, M.A., M.R.C.P., of Bradford, has been appointed Physician to the Sheffield Hospital and Dispensary.

The annual meeting of the Governors of the Taunton and Somerset Hospital was held on Tuesday afternoon, under the presidency of T. M. Dodington, Esq., High Sheriff. The Chairman mentioned that a munificent bequest of £500 by Lady Clarges had saved the Institution from serious financial difficulty.

The matron of the Liverpool Eye and Ear Infirmary writes to a local paper, asking contributions of underclothing for children, many of whom, it is stated, suffer great hardships in consequence of being insufficiently clothed. The inhabitants of Liverpool and the neighbourhod only need to be informed in order to supply all that is required.

The Managers of the Manchester Royal Infirmary have, by resolution, decided to decline the offer made to them by Mr. J. W. Maclure, M.P., to erect and equip a hospital for twenty beds in the town of Hulme, to be managed and maintained by them.

Three hundred red flannel caps lined with calico are asked for on behalf of the patients of the Newcastle Infirmary. It is found that many hospital patients suffer from colds in the head, in consequence of the necessity for opening the windows, near which their beds are often placed.

The children of the Frederick Street Industrial School have sent a contribution of £1 4s. to the Consumption Hospital at Belfast. On their behalf Miss B. Whyte writes: "The children have requested me to give you the enclosed £1 4s. for the Consumption Hospital, being part of the proceeds of their little sale of work. The sum is small, but the children are very poor, and it only to serves to show their deep sense of the terrible need for an institution where the poor consumptive patients may be made comparatively comfortable in the last moments of their existence. Hoping this may be lucky, and that large sums from the rich will pour in speedily, and as heartily."

It appears probable that the Hospital Sunday collections at Birmingham this year will be slightly in excess of the amounts taken last year for the same purpose. Among the larger amounts contributed, we are glad to see a sum of £150 by the Hebrew Synagogue. In spite, however, of this increase, it is doubtful if the total amount will touch the highest point reached in a previous year.

The *Newcastle Chronicle* says: "For some years past on each Hospital Sunday a friendly rivalry has existed between Jesmond Parish Church and Brunswick Place Wesleyan Chapel, Newcastle, as to which should contribute the larger amount to the fund. This year the parish church heads the list with collections amounting to £104 2s. 4d.

Not only the managers, secretaries, and matrons of medical institutions, but their supporters also, find themselves in a difficulty when they desire to procure accurate information upon certain matters affecting their management. The Hospitals Association has been quietly organising a system to meet this want. Formerly there was no course open to the inquirer but to address a circular letter to a certain number of selected institutions, asking for information upon the matters at issue. Many will therefore welcome the announcement that a system has been organised, and an amount of information collected by the Hospitals Association, which will secure a prompt, accurate, and exhaustive reply to questions which may be addressed to the Secretary, Norfolk House, Norfolk Street, Strand, W.C. This Association deserves well of the medical institutions, their officers, and the members of the public who support them. It is actively working to increase the amount of voluntary support given to all the hospitals; it is organising a National Pension Fund for nurses and hospital officials; it has opened a register for trained nurses; and to these is now added an inquiry office, where anyone interested in hospitals may apply for information with the certainty of getting to know exactly what they want without unnecessary delay or difficulty.

Up to the present time a little over £1,000 has been sent in as the result of the Hospital Sunday collections at Brighton.

The *East Anglian Daily Times* says: "We understand that differences have arisen at the East Suffolk and Ipswich Hospital which have led Mr. Montague, the house surgeon, to tender his resignation. Mr. Montague was elected about two months ago, his predecessor having resigned under exactly similar circumstances. Mr. Montague is the third house surgeon elected by the Board of Management who has resigned since Christmas." Can any reader explain what is the cause of this disorganization?

The charges made against Mr. O'Grady, the senior surgeon at Mercers' Hospital, Dublin, are now the subject of inquiry by the authorities of the institution.

The Rev. Canon Machell, preaching on behalf of the Hospital Sunday Fund, at Hull, said: "There was no moral wrong in one man living in a palace and another in a cottage, but there would be a moral wrong if the cottage was undrained, unhealthy, unfit for human habitation; if there was no provision made for decency, if there was nothing there which would promote the means of living a true life; if there were no sanitary equality between them. Why did we still serve with so much love and affection a Queen who had reigned in the hearts of her people for fifty years? Because during that half-century we believed it had been her great aim to be good herself and to do good to others. He would not advocate the extravagant expenditure of money in either pomp or show to celebrate the Jubilee, but he would advocate on an occasion like the present to mark this year above all others as one when men gave of their abundance; gave liberally—even more liberally than they ever gave before—to the great institutions for which he was then pleading."

A contributor to the *Ladies' Pictorial* says: "I sincerely hope that the statement made by a correspondent in a Bristol paper to the effect that a well-known judge, some short time ago, was treated at a London Hospital and forgot to make a donation, is untrue. I am afraid, however, that a very considerable amount of meanness is to be found amongst many of those who take advantage of the benefits of a free hospital system. That, however, a learned judicial light, and of course comparatively-speaking wealthy man, should descend to such a paltry little trick is all but past belief."

It is gratifying to be able to report that the amount collected in London on Hospital Sunday, in June last, was £40,605 4*s*. 2*d*., the largest sum ever obtained for the hospitals by this fund. All honour to H.R.H. the Duke of Cambridge, the Marquis of Salisbury, the Earl of Rosebery, Baron Rothschild, M.P., and the other eminent men who lent the weight of their influence to aid the hospitals at a critical time in their history, and so secured this result. The smallest sum ever collected in London on Hospital Sunday was £24,905, in 1878. The fund increased from £26,501 in 1879, to £30,441 in 1880, and thenceforth steadily up to £39,330 in 1884, when a special gift of some thousands of pounds was received from a fête at the Healtheries. The fund fell again to £34,320 in 1885, and has been raised to its present amount by the exertions of those members of the Council who are responsible for the institution of The Hospitals Week. These results have fully justified the new departure taken by the Council in 1886, and should secure the co-operation of the managers of all the hospitals to raise the fund to £50,000 at least during 1888.

Dr. Walter S. A. Griffiths, M.R.C.P., F.R.C.S., has been appointed obstetric physician for in-patients to the Great Northern Central Hospital, in place of Gustavus C. P. Murray, M.D., deceased.

The Jubilee Fund now being raised in the parish of Islington on behalf of the new buildings of the Great Northern Central Hospital already amounts to £4,000. The history of this fund well illustrates the oft-repeated statement that hospitals derive their support from an exceedingly limited number of persons, compared with that of the whole population. It is said that the £4,000 has been subscribed entirely by 600 donors, although the parish of Islington alone contains a population of considerably over 300,000. To the 600 subscribers, too much praise can hardly be given for the average amount of their donations, but to the remaining inhabitants of the parish, bearing to the subscribers the proportion of nearly 600 to 1, the entire absence of sympathy and help for so important a movement is nothing short of disgraceful.

The fever epidemic, we regret to say, shows but small indications of diminution. The numbers removed to the hospitals of the Asylums Board from day to day preserve about the same average. It may be hoped that when the present damp and unhealthy atmospheric conditions pass away the abatement will be speedy and permanent.

The Dean of Gloucester, preaching on behalf of the Cheltenham General Hospital at Christchurch on Sunday morning, said: "There were those who said that the management of the hospital was bad. Let the hospital critics in Cheltenham and elsewhere show them another and better way if they could. Till then let them help with heart and soul and with self-denial."

At St. Paul's Church, Cheltenham, the Rev. W. H. Wright spoke from an opposite point of view to that of the Dean. He, after freely criticising several circumstances in connection with the hospital, said "a plate would be held at the door of the church to enable those who wished to contribute to do so, but he would not have the plate forced upon anyone. He himself was not a subscriber; they might give if they chose. They knew their pastor's views on the subject."

The authorities of the Scarborough Hospital and Dispensary are appealing for a supply of old linen. Many such appeals may now be seen in the local papers, and it will be well if housewives in setting their houses in order annually would make a point of putting aside their old linen for hospital purposes.

